# Prediction of malignant transformation in oral epithelial dysplasia using machine learning

**DOI:** 10.1088/2633-1357/ac95e2

**Published:** 2022-10-07

**Authors:** James Ingham, Caroline I Smith, Barnaby G Ellis, Conor A Whitley, Asterios Triantafyllou, Philip J Gunning, Steve D Barrett, Peter Gardener, Richard J Shaw, Janet M Risk, Peter Weightman

**Affiliations:** 1 Department of Physics, University of Liverpool, L69 7ZE, United Kingdom; 2 Department of Pathology, Liverpool Clinical Laboratories, University of Liverpool, L69 3GA, United Kingdom; 3 Department of Molecular and Clinical Cancer Medicine, Institute of Systems, Molecular and Integrative Biology, University of Liverpool, L3 9TA, United Kingdom; 4 Manchester Institute of Biotechnology, University of Manchester, M1 7DN, United Kingdom; 5 Regional Maxillofacial Unit, Liverpool University Hospitals NHS Foundation Trust, Liverpool, L9 7AL, United Kingdom

**Keywords:** machine learning, oral cancer, OED, FTIR spectroscopy

## Abstract

A machine learning algorithm (MLA) has been applied to a Fourier transform infrared spectroscopy (FTIR) dataset previously analysed with a principal component analysis (PCA) linear discriminant analysis (LDA) model. This comparison has confirmed the robustness of FTIR as a prognostic tool for oral epithelial dysplasia (OED). The MLA is able to predict malignancy with a sensitivity of 84 ± 3% and a specificity of 79 ± 3%. It provides key wavenumbers that will be important for the development of devices that can be used for improved prognosis of OED.

## Introduction

Fourier transform infrared (FTIR) spectroscopy is increasingly being used in a wide variety of research areas and has demonstrated a significant potential in cancer related studies [1]. It has been shown that it is possible to classify oral squamous cell carcinoma (OSCC) by analysing FTIR spectral images [2], and other studies have successfully associated vibrational spectroscopy data with histopathological classification of potentially malignant oral lesions [3, 4]. A recent study [5] used a supervised, retrospective analysis of tissue samples from patients with high-risk oral epithelial dysplasia (OED) lesions. As these archival samples came with prolonged, clinical follow-up and known outcomes (transformation or no transformation to cancer), it was possible to train and test a principal component analysis (PCA) linear discriminant analysis (LDA) model for the prediction of malignant transformation for OED. This model was able to predict malignancy with a sensitivity of 79 ± 5% and specificity of 76 ± 5% and highlighted six key wavenumbers needed for accurate discrimination. In order to test the robustness of this analysis, the dataset has now been analysed with a different approach using a novel machine learning algorithm (MLA) [6].

## Methods

This study utilised samples from seventeen patients with biopsy-proven OED. The collection, preparation and classification of samples has been described previously [5]. All patients have given written informed consent to a UK NHS Research Ethics Committee approved study that was carried out in compliance with the Helsinki Declaration (Liverpool Central REC ref: EC 47.01). Samples were divided into two sets based solely on the known clinical outcome of the lesion: T (lesions underwent malignant transformation, n = 10) and NT (lesions did not undergo transformation, n = 7).

The collection and processing of FTIR spectral images has also been described previously [5] and these results are re-analysed in this work using a supervised machine learning algorithm (MLA) that was developed for the characterisation of oesophageal cancer cells [6] and recently applied to the characterisation of metastatic oral cancer tissue [2].

The approach taken by the MLA is described in detail in [6], but to provide some context we outline here a summary of the key methodology. The MLA comprises three stages; training, testing and validation. Within the training stage distributions of values of absorbance ratios at two wavenumbers are used in an attempt to discriminate various tissue types. All pairings of wavenumbers and their corresponding distributions are defined in the MLA as ‘metrics’. The testing stage quantifies the ability of each metric to discriminate between each tissue type using half of the remaining spectral datasets not used in the training stage. The second phase of the testing stage compiles an output containing high scoring metrics that are best able to discriminate all the tissue types. The validation stage uses these metrics to label spectra not used in any of the previous training or testing stages. Knowing the actual tissue type of these labelled spectra, values of sensitivity and specificity can be determined.

The MLA, available from the authors upon request, was implemented in MATLAB. The dataset was divided randomly into training (60%), testing (20%) and validation (20%) sets. When this was done the T and NT were treated as two separate subsets. In order to assess the reliability of the results this process was repeated ten times, each time with the training and testing subsets re-randomised. Pre-processing of the dataset was carried out as described in [6].

## Results

The performance of the MLA and the PCA-LDA study [5] are summarised in table 1. The two approaches yield very similar results with the MLA showing a slight improvement over the PCA-LDA in terms of sensitivity, specificity and precision. This confirms the efficacy of FTIR analysis to discriminate between samples with similar histology but different outcome. The improvement in the performance of the MLA over the PCA-LDA is not as important as the overall agreement between the two approaches.

**Table 1.** Overall performance of the MLA and PCA-LDA.

	MLA	PCA-LDA
Sensitivity	84 ± 3%	79 ± 5%
Specificity	79 ± 3%	76 ± 5%

**Figure 1.**
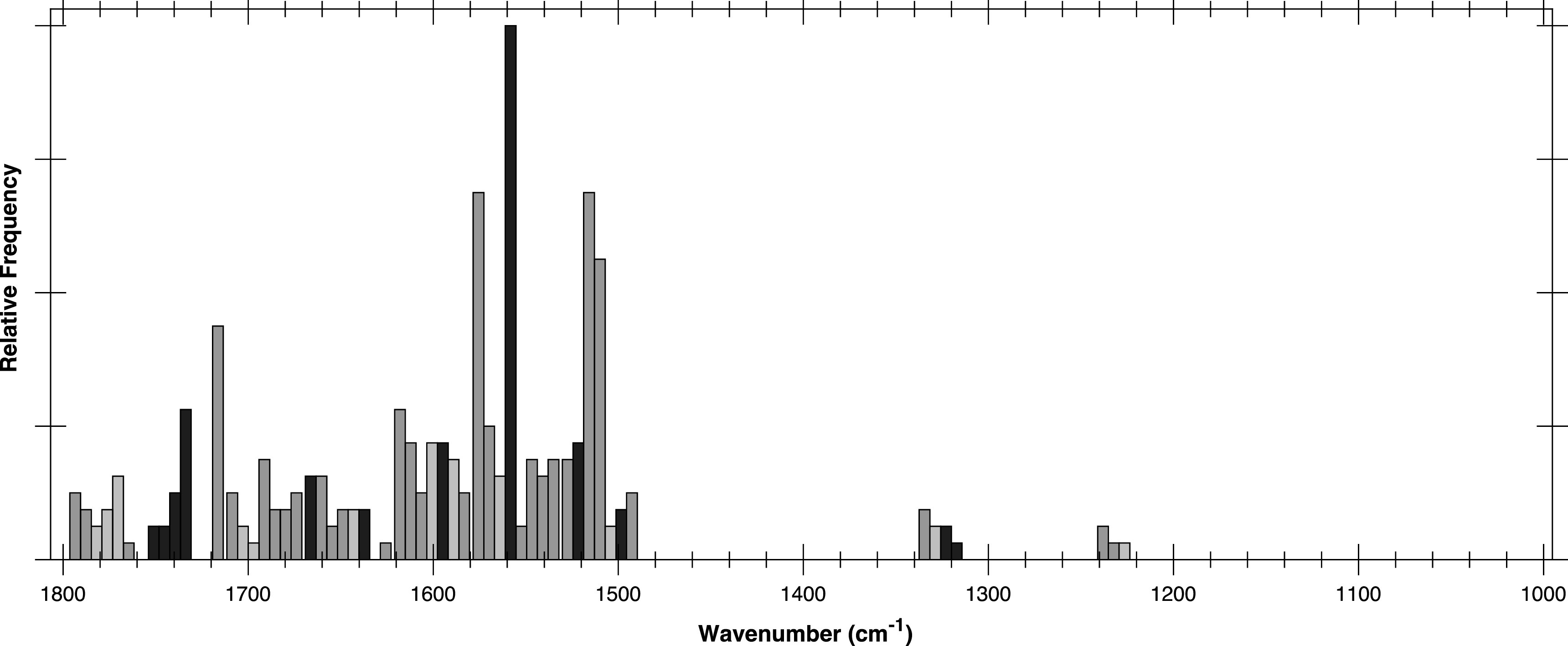
Histogram showing how frequently discriminating wavenumbers appear in the MLA for the top 150 metrics. Green indicates wavenumbers corresponding to important (positively or negatively weighted) features in the PCA-LDA; blue corresponds to regions of the spectrum which are neutral in the PCA-LDA; wavenumbers that fall into neither of these categories are shown in grey. MLA wavenumbers are considered to in correspondence with PCA-LDA wavenumbers if they are within the spectral resolution.

Figure 1 shows a histogram of the most important wavenumbers found by the MLA, colour-coded according to their correlation with wavenumbers found in the PCA-LDA. The wavenumbers indicated in green correspond to important (positively or negatively weighted) features in the PCA-LDA. The wavenumbers indicated in blue correspond to regions of the spectrum which are neutral in the PCA-LDA analysis. Wavenumbers that fall into neither of these categories are shown in grey. In contrast to PCA-LDA, the neutral wavenumbers can play an important role in the MLA. They act as a reference when taking ratios of intensities and can mitigate against systematic errors arising from sample-to-sample variations [6]. The small fraction of wavenumbers shown in grey in figure 1 indicate that there is a very strong correlation between the wavenumbers identified in the MLA and the features that are important in the PCA-LDA.

Table 2 shows a quantitative comparison between the wavenumbers found by the two approaches. For the MLA, the weighted averages of distinct groups of neighbouring wavenumbers (green) are shown.

**Table 2.** Comparison of key wavenumbers (in cm^−1^) identified in the MLA and PCA-LDA.

MLA	PCA-LDA
1684	1678
1656	1653
1614	1628
1575	1574
1236	1242
—	1020

There is very good agreement between the key wavenumbers identified by the two different approaches. In four of these comparisons the differences are comparable to the spectral resolution of 4 cm^−1^. The apparent discrepancy between 1614 cm^−1^ and 1628 cm^−1^ arises because the feature identified in the PCA-LDA analysis is distinctly asymmetric (see figure 3(c) of [5]) and this skews the value to a higher wavenumber. There is no corresponding entry against the PCA-LDA value of 1020 cm^−1^ because this wavenumber is not found in the top 150 metrics (equivalent to 0.3% of all metrics calculated). This is probably due to this region of the spectrum being particularly sensitive to the details of the pre-processing employed in the PCA-LDA analysis.

## Discussion

The strengths and weaknesses of these two approaches to the analysis of cancerous cells and tissue are explained in [2], [5] and [6]. The two approaches can be assessed from two complementary viewpoints. The first is the clinical aspect, which requires the highest sensitivities and specificities possible for accurate prognosis and better patient treatment. The other aspect is the scientific insight gained into the underlying mechanisms involved in cancer.

The performances of both models were comparable, with the MLA slightly outperforming the PCA-LDA. Both approaches result in substantial improvements over the 40% obtained from current gold standard techniques [7]. The parity in scores may indicate that both approaches are close to optimal.

The two approaches both clearly identify a number of key wavenumbers and there is significant overlap between the two sets of results (table 2). We note that many of these wavenumbers occur in the Amide I and Amide II regions. It is beyond the scope of this paper to interpret the chemical and biological significance of these specific IR biomarkers. However, the interpretation of these biomarkers has clear potential for obtaining an insight into chemical processes taking place in early cancer and this will be explored in future studies. In addition, it is anticipated that this approach could be translated into clinical applications.

## Conclusions

The most important result of this analysis is that two very different approaches have established that the analysis of FTIR spectra can predict which OED lesions will become malignant. Both approaches have a predictive accuracy of ∼80% which has important implications for diagnosis and therapy. Current approaches are only able to make this prediction with an accuracy of 25%–40% [7] and so fail 60% of patients. This leads to over treatment, requiring unnecessary and painful biopsies, or under treatment resulting in delays to necessary surgery.

The key wavenumbers obtained by the MLA, and in particular the reference wavenumbers (shown in blue in figure 1) not found by the PCA-LDA, will facilitate the development of devices for the early prognosis of OED [8]. Moreover, the MLA may have considerable diagnostic potential when applied to other diseases where clinical/pathological discrimination of subtle, early changes is difficult.

## Data Availability

The data that support the findings of this study are openly available at the following URL/DOI: https://doi.org/10.17638/datacat.liverpool.ac.uk/1622.
